# Resistance to anti-PD-1-based immunotherapy in basal cell carcinoma: a case report and review of the literature

**DOI:** 10.1186/s40425-018-0439-2

**Published:** 2018-11-20

**Authors:** Francesco Sabbatino, Antonio Marra, Luigi Liguori, Giosuè Scognamiglio, Celeste Fusciello, Gerardo Botti, Soldano Ferrone, Stefano Pepe

**Affiliations:** 1 Medical Oncology Unit, Department of Medicine, Surgery and Dentistry, University of Salerno, 84131 Salerno, Italy; 2 Medical Oncology Unit, ASST Santi Paolo e Carlo, University of Milan, Milan, Italy; 3 Department of Pathology, Istituto Nazionale Tumori – IRCCS- Fondazione G. Pascale, Naples, Italy; 4 Department of Surgery, Massachusetts General Hospital, Harvard Medical School, Boston, MA USA

**Keywords:** Basal cell carcinoma, Immunotherapy, PD-1, PD-L1, HLA class I antigens, β2-microglobulin, Immune escape, Regulatory immune cells, T cell infiltration, Nivolumab

## Abstract

**Background:**

Immunotherapy with immune checkpoint inhibitors has radically changed the management of a broad spectrum of tumors. In contrast, only very limited information is available about the efficacy of these therapies in non-melanoma skin cancers, especially in basal cell carcinoma. The latter malignancy is often associated with both an impairment of the host immune response and a high mutation burden, suggesting that immune checkpoint inhibitor-based immunotherapy may be effective in the treatment of this tumor.

**Case presentation:**

A 78-year-old woman was diagnosed with a metastatic non-small-cell-lung-cancer. Following the lack of response to two lines of systemic chemotherapy, she was treated with the anti-PD-1 monoclonal antibody nivolumab, obtaining a prolonged stable disease. Under nivolumab treatment, the patient developed a basal cell carcinoma of the nose. The latter was surgically resected. Immunohistochemical staining of tumor tissue showed a PD-L1 expression < 1% and lack of human leukocyte antigen class I subunit (i.e. heavy and light chain) expression on tumor cells. In addition, a limited number of T cells (CD3+) was present in the tumor microenvironment, with a higher number of regulatory T cells (Foxp3+) and macrophages (Cd11b+) as compared to a low infiltration of activated cytotoxic T cells (CD8+/ Granzyme B+). Two months following the surgical removal of the tumor, while still on nivolumab treatment, the patient relapsed with a basal cell carcinoma in the same anatomic site of the previous surgical excision. The tumor displayed the same pathological characteristics.

**Conclusion:**

Preclinical lines of evidence suggest a potential role of immune checkpoint inhibitors for basal cell carcinoma treatment. However, limited clinical data is available. In the patient we have described administration of the immune checkpoint inhibitor nivolumab for the treatment of a responsive non-small cell carcinoma was associated with the development and relapse of a basal cell carcinoma tumor. This association is likely to reflect the resistance of basal cell carcinoma cells to anti-PD-1 based immunotherapy because of a “cold” tumor microenvironment characterized by lack of human leukocyte antigen class I expression, low PD-L1 expression and high number of immune regulatory cells.

## Background

Basal cell carcinoma (BCC) is the most common human cancer, accounting for about 25% of all diagnosed tumors worldwide [1, 2]. Although BCC can be often controlled by radical surgery, it can present aggressive features such as local recurrence, tissue destruction and in a small percentage of cases widespread dissemination [3, 4]. A deeper knowledge of the mechanisms underlying BCC development and progression has allowed the discovery of mutations in the sonic hedgehog homolog (SHH) pathway as the most common oncogenic alterations [5, 6]. These observations have led to the use of small molecules targeting the SHH pathway such as vismodegib and sonidegib, both currently approved for the treatment of recurrent or metastatic BCC [7–9]. However, the efficacy of these agents is limited due to the progressive development of drug resistance [10, 11] emphasizing the need to develop novel therapeutic agents. Recently, immune checkpoint inhibitors (ICIs) such as anti-programmed death-1 (PD-1)- and -programmed death-ligand 1 (PD-L1) monoclonal antibodies (mAbs) have markedly changed the treatment of several types of cancer, significantly improving patient survival and quality-of-life [12]. For example, in non-small-cell lung cancer (NSCLC), ICIs have shown to be effective in first and advanced lines of the metastatic setting [13] as well as in the locally advanced NSCLC improving the overall response rate (ORR), progression-free survival (PFS) and overall survival (OS) of treated patients as compared to standard chemotherapy [14–21]. In contrast ICIs are still in the early stages of clinical assessment for treating BCCs and limited clinical evidence is at present available about their therapeutic efficacy [22–26]. Furthermore ICI-based immunotherapy is effective only in a small subset of cancer patients and no clear predictive biomarker of response has been identified so far.

Here, first, we will describe a patient who developed a BCC during treatment of metastatic NSCLC with the anti PD-1 mAb nivolumab. Second, we will analyze and describe the potential mechanisms of tumor immune escape developed by BCC cells that are associated with lack of BCC response to nivolumab. Lastly we will discuss the most relevant lines of clinical evidence utilizing ICIs for the treatment of BCC patients and the predictive biomarkers identified in order to select BCC patients who are more likely to benefit from this type of therapy.

## Case presentation

**Fig. 1:**
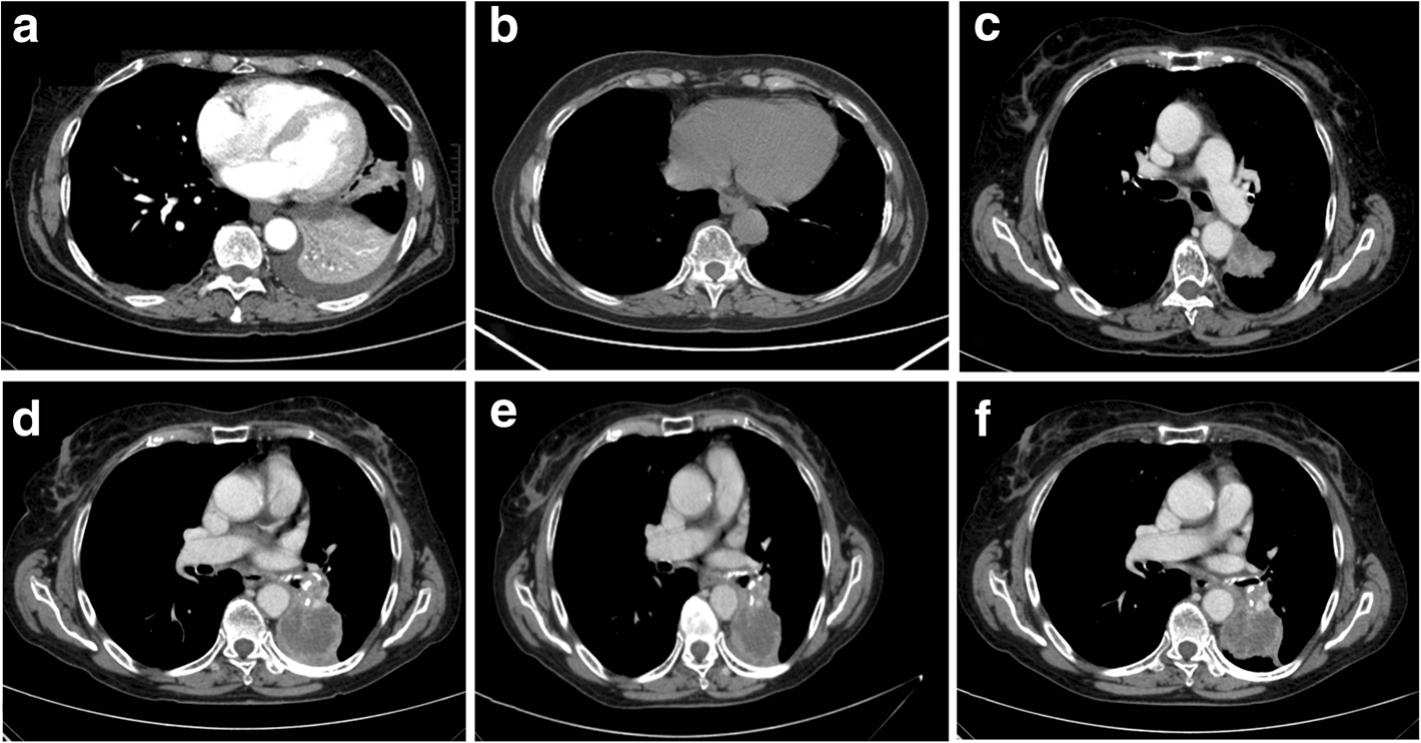
Chest CT-scan performed at diagnosis in October 2013 (**a**), in May 2014 following first line chemotherapy (**b**), in April 2015 at tumor progression following radiotherapy (**c**), in January 2016 before starting immunotherapy (**d**), in May 2016 during immunotherapy (**e**) and in December 2017 following 38 cycles of immunotherapy administration (**f**)

In October 2013, a 78-year-old woman was admitted to our Oncology Unit because of the development of costal pain and cough. A chest CT-scan showed a complete atelectasis of the left lung inferior lobe, a solitary nodule in apex segment of the upper right lung lobe, the presence of multiple small nodules in basal posterior segments of the right lung lobe as well as in the middle and upper left lung lobes, and a left pleural effusion (Fig. 1a). She had a good performance status (PS) (ECOG PS = 0). Her prior medical history was remarkable for surgical excisions of two nodular BCCs of the trunk (0.5 × 0.3 cm and 0.6 × 0.7 cm, respectively, both without perivascular and perineural invasion) in 2003. In addition, she was an active smoker (40 packs/year).

In order to complete the pathological and clinical staging, the patient received a whole-body ^18^FDG-PET/CT and a bronchoscopy with a biopsy of the left lung lesion. The ^18^FDG-PET/CT showed a high metabolic activity of the lesion in the left lung, whereas the other lesions did not show any metabolic activity. The pathological examination demonstrated the diagnosis of lung squamous cell carcinoma (p63+, TTF-1-). She was staged as a stage IV NSCLC (according to TNM staging 7th edition).

Because of her good clinical conditions, the patient was treated with six cycles of chemotherapy with gemcitabine (1250 mg/m^2^), on days 1 and 8, plus cisplatin (75 mg/m^2^) on day 8, every 21 days. In May 2014, whole-body CT scan showed a partial response (PR) (according to RECIST 1.1 criteria) following six cycles of chemotherapy with 90% reduction of the hilar lesion and disappearance of the pleural effusion (Fig. 1b). Following multidisciplinary discussion, the patient received a consolidative radiotherapy treatment on the residual disease, obtaining a stable disease (SD) for an additional 8 months. However, in April 2015, a whole-body CT scan showed a progression of disease (PD) with an increased diameter of the left hilar lesion and the appearance of several hilar lymph nodes (Fig. 1c). Thus the patient received a second-line chemotherapy with 6 cycles of docetaxel (75 mg/m^2^) every 21 days, obtaining a SD. Unfortunately, in January 2016, a whole-body CT scan showed a PD with an increase of the pulmonary hilar lesion associated with atelectasis of the inferior left lobar bronchus and several pathological mediastinal lymph nodes (Fig. 1d). In order to reanalyze tumor histology and the molecular profile, we decided to perform a re-biopsy of the left lung lesion. Pathological examination confirmed the diagnosis of lung squamous cell carcinoma. No targetable oncogenic alterations (EGFR mutations, ALK/ROS-1 rearrangements and BRAF mutations) were detected. PD-L1 expression on tumor cells was scored as > 1% on tumor proportional score (TPS).

**Fig. 2:**
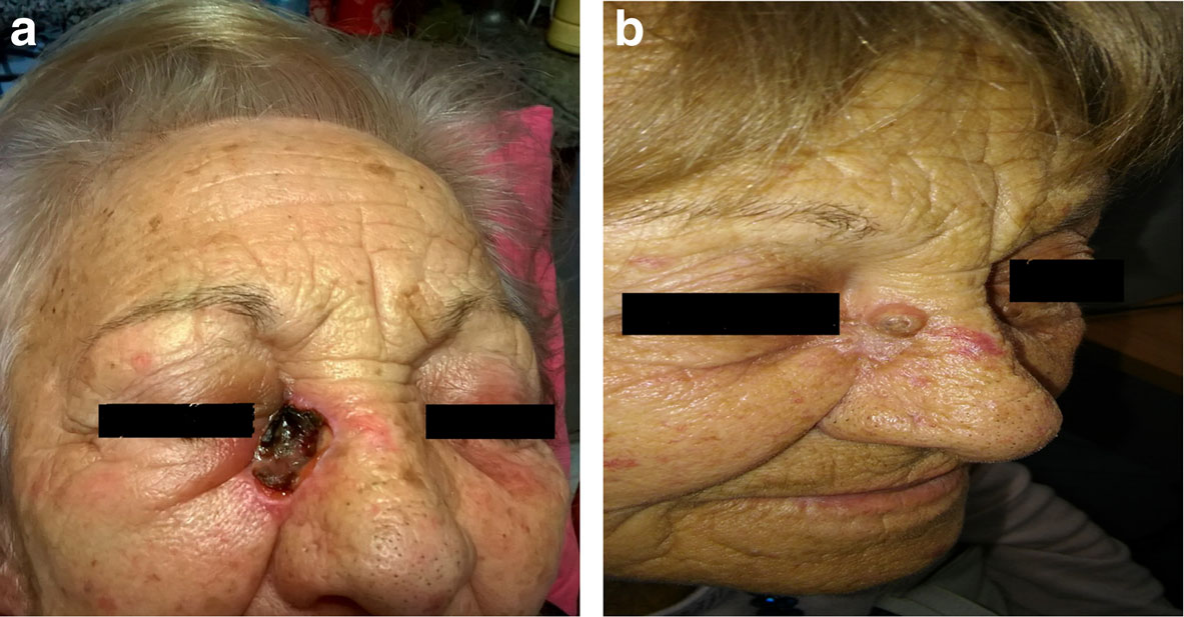
**a** Primary BCC developed by the patient during nivolumab treatment. **b** BCC relapse after surgery

**Fig. 3:**
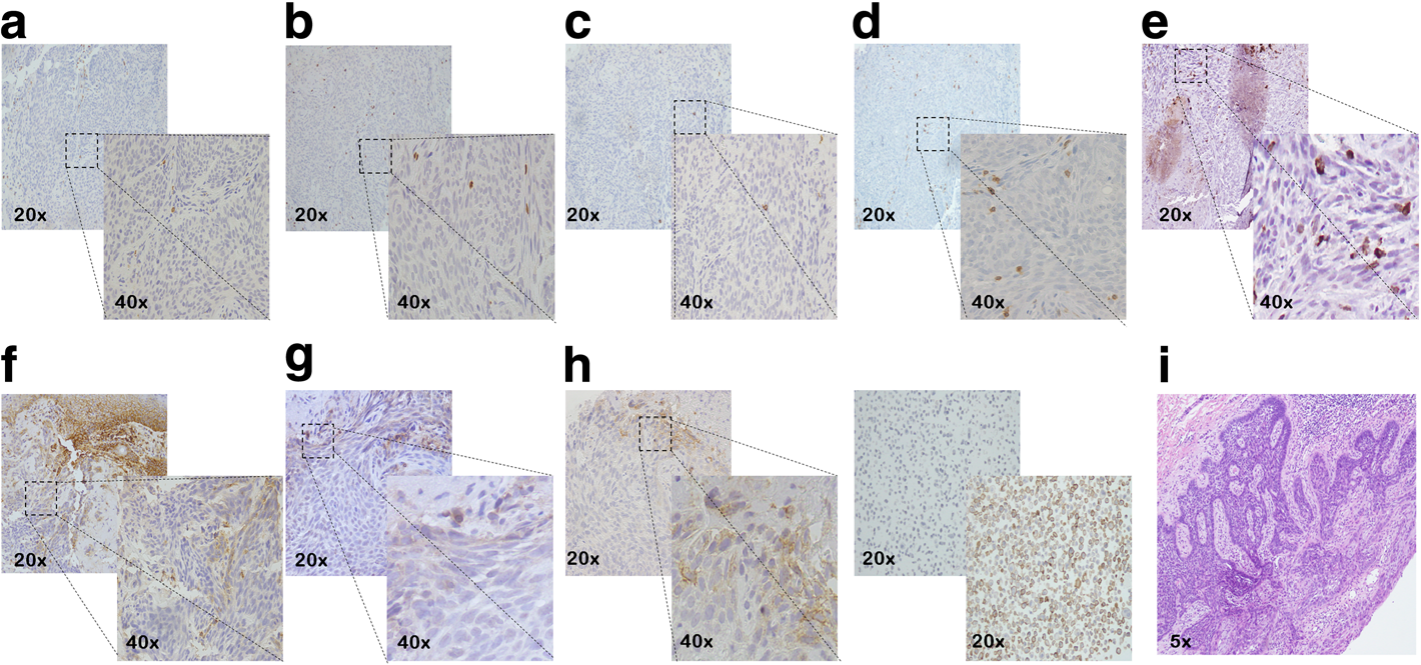
Representative staining patterns of the formalin-fixed, paraffin-embedded primary BCC lesion with granzyme-B (**a**), FOXP3 (**b**), CD8 (**c**), CD3 (**d**) and CD11b (**e**) specific mAbs. Number of positive cells were enumerated in an entire lesion and reported as an absolute number. **f** Representative staining patterns of the formalin-fixed, paraffin-embedded primary BCC lesion with HLA class I antigen-specific mAbs. Tumor tissue sections were immunohistochemically (IHC) stained with a pool of mouse HLA-A–specific mAb HCA2 and HLA-B/C-specific mAb HC10 (ratio, 1:1). mAb HCA2 recognizes β2m-free HLA-A (excluding -A24), -B7301, and -G heavy chains; mAb HC10 recognizes β2m-free HLA-A3, −A10, −A28, −A29, −A30, −A31, −A32, −A33, and all β2m-free -HLA-B (excluding -B5702, -B5804, and -B73) and -HLA-C heavy chains [53–55]. IHC staining was performed as described previously [56]. Staining of infiltrating immune cells was used as an internal positive control. The staining with HLA class I antigen–specific mAbs on tumor cells was scored as negative because HLA class I antigen score in an entire lesion was 0. **g** Representative staining patterns of the formalin-fixed, paraffin-embedded primary BCC lesion with β2m-specific mAb NAMB-1 [57]. IHC staining was performed as described previously [56]. Staining of infiltrating immune cells or fibroblasts was used as an internal positive control. The staining with β2m-specific mAb on tumor cells was scored as negative because β2m score in an entire lesion was 0. **h** Representative staining patterns of the formalin-fixed, paraffin-embedded primary BCC lesion with PD-L1-specific mAb (left panel). PD-L1 IHC staining was performed utilizing the automated PD-L1 IHC assay (PD-L1 IHC 28–8 pharmDx SK005) with Dako’s Autostainer Link 48 [58]. This test is a complementary diagnostic for nivolumab. MCF7 and NCI-H226 cell lines were used as a negative (upper and right panel) and positive control (bottom and right panel), respectively, accordingly to manufacturer kit instructions. PD-L1 expression was scored as negative because PD-L1 score in an entire lesion was < 1%, both on tumor cells and immune cells. Slides were reviewed and enumerated by an experienced pathologist (GB). **i** Representative staining patterns of the formalin-fixed, paraffin-embedded primary BCC lesion with hematoxylin and eosin (H&E). Magnification is indicated

Based on these results, we decided to start a third-line treatment with the anti-PD-1 mAb nivolumab at the dose of 3 mg/kg, every 14 days. In February 2016, the patient started the administration of nivolumab. In May 2016, a CT scan showed a SD (Fig. 1e) which was confirmed in successive restaging of the disease (Fig. 1f). Following 18 cycles of nivolumab treatment (in January 2017), in a good performance status and without experience of any immune-related adverse event, the patient developed an ulcerated lesion (diameter = 1.0 × 1.4 cm) localized at the right ala of nose (Fig. 2a). While she was on nivolumab treatment, in February 2017, she underwent an excisional skin biopsy. Histological examination of the lesion showed an ulcerated nodular BCC. Perivascular and perineural invasions were not detected. Surgical tumor margins were negative. Immunohistochemical (IHC) staining of tumor showed a PD-L1 expression < 1%, on both tumor cells and immune cells, and lack of human leukocyte antigen (HLA) class I and β2-microglobulin (β2m) expression on tumor cells (Fig. 3). In addition, a limited number of T cells (CD3+) was present in the tumor microenvironment, with a higher number of regulatory T cells (Foxp3+) and macrophages (CD11b+) as compared to activated cytotoxic T cells (CD8+/ Granzyme B+) (Fig. 3). After two months, while still being treated with nivolumab, the patient relapsed with a new BCC lesion in the same region of the previous surgical excision (Fig. 2b). A new surgical excision was performed and the pathological examination confirmed the diagnosis of a relapsed BCC with same pathological characteristics.

At present, the patient is still being treated with nivolumab. She has received 46 cycles of nivolumab. Sustained stable disease control is still ongoing for metastatic NSCLC. She is in good health conditions. No treatment-related toxicities have been observed. No additional BCC relapses have been detected so far.

## Discussion

Over the last ten years, the implementation of ICI-based immunotherapy has been one of the major breakthroughs for the treatment of cancer patients. Several mAbs targeting immune checkpoint molecules such as Cytotoxic T Lymphocyte Antigen-4 (CTLA-4), PD-1 and PD-L1 have been approved for the treatment of a broad spectrum of cancers [12]. ICIs are also currently being investigated for patients with relapsed-recurrent or metastatic BCC (Table 1). However the clinical efficacy of ICIs is limited to a small percentage of treated patients. The identification of predictive biomarkers of response to ICIs represents at present one of the major challenges in cancer research [27, 28]. PD-L1 expression has been the most explored predictive biomarker so far. Several studies have shown a significant correlation between PD-L1 expression in the tumor microenvironment and an increased likelihood of response to anti-PD-1/PD-L1 therapy [19, 29]. In contrast, many other studies have also shown that patients who do not express PD-L1 in the tumor microenvironment may also benefit from anti-PD-1/PD-L1-based immunotherapy [14–18]. Overall, PD-L1 is considered a “surrogate” biomarker that can be used to predict patients who are more likely to benefit from anti-PD-1/PD-L1 immunotherapy. In the patient we have described administration of nivolumab for the treatment of a responsive NSCLC was associated with the development and relapse of a BCC tumor. Several lines of evidence have highlighted the critical role of immune surveillance in the control of BCC, as demonstrated by the increased incidence of these tumors in immunosuppressed subjects [30, 31].

**Table 1:** Ongoing clinical trials testing immunotherapeutic agents in BCC patients

Agent	Phase	Condition	Intervention	Status	ClinicalTrials.gov ID
Talimogene Laherparepvec	I	Non-melanoma Skin Cancer	Evaluation of the mechanism of action of talimogene laherparepvec (T-VEC) in patients with locally advanced non-melanoma skin cancer	Not yet recruiting	NCT03458117
		Basal Cell Carcinoma			
		Squamous Cell Carcinoma Cutaneous Lymphoma			
		Merkel Cell Carcinoma			
ASN-002	I/II	Basal Cell Carcinoma in Basal Cell Nevus Syndrome	Study of ASN-002 to Treat Basal Cell Carcinomas in Individuals with Basal Cell Nevus Syndrome	Not yet recruiting	NCT03208296
REGN2810	II	Basal Cell Carcinoma	Anti-PD-1 in Patients with Advanced Basal Cell Carcinoma Who Experienced Progression of Disease on Hedgehog Pathway Inhibitor Therapy, or Were Intolerant of Prior Hedgehog Pathway Inhibitor Therapy	Recruiting	NCT03132636
Nivolumab + Talimogene Laherparepvec	II	Refractory Lymphomas Advanced or Refractory Non-melanoma Skin Cancers	Talimogene Laherparepvec and Nivolumab in Treating Patients with Refractory Lymphomas or Advanced or Refractory Non-melanoma Skin Cancers	Recruiting	NCT02978625
Pembrolizumab +/− Vismodegib	II	Skin Basal Cell Carcinoma	Pembrolizumab With or Without Vismodegib in Treating Metastatic or Unresectable Basal Cell Skin Cancer	Active, not recruiting	NCT02690948
ASN-002 +/− 5-FU	I/II	Basal Cell Nevus Syndrome Skin Neoplasm Nodular Basal Cell Carcinoma of Skin	A Study of the Efficacy and Safety of ASN-002 in Adult Patients with Low-risk Nodular Basal Cell Carcinoma	Recruiting	NCT02550678

In order to identify the potential mechanisms underlying nivolumab inability to control BCC development, we analyzed the expression of PD-L1 in both the primary and the relapsed tumor. Conflicting data about PD-L1 expression in BCC has been reported in the literature so far. Chang et al. analyzed 138 BCCs showing a PD-L1 expression on tumor cells and tumor infiltrating lymphocytes (TILs) of 89.9 and 94.9%, respectively. More importantly, PD-L1 expression was higher in previously treated patients as compared to treatment-*naïve* subjects [32]. In contrast, Lipson et al. analyzed 40 BCCs and showed a PD-L1 expression on tumor cells and TILs of 22.0 and 82.0%, respectively [23]. The same authors also described a previously-treated BCC patient who carried a tumor with a high PD-L1 expression. The patient was treated with the anti-PD-1 mAb pembrolizumab and obtained a prolonged PR [23]. Similar results have been reported by Falchook et al. [24] and by Winkler et al. [33]. The latter investigators showed that a metastatic BCC patient obtained a SD following pembrolizumab administration [33]. The former investigators reported that a previously-treated BCC patient obtained a partial response that lasted more than 12 months following treatment with the anti-PD-1 mAb cemiplimab [24]. In the latter two patients, PD-L1 expression was non-detectable [24] or low [33]. Our treatment-naïve BCC patient, who expressed PD-L1 on < 1% of both tumor cells and TILs, did not respond to anti-PD-1 immunotherapy. These conflicting results most likely reflect the different mAbs used to detect PD-L1 expression and the PD-L1 cut-off adopted, and/or the different characteristics of the BCC tumors in the three patients investigated.

Besides PD-L1, other predictive biomarkers for ICIs have been investigated or are currently under evaluation. Among all tested markers, the presence of a high tumor mutational burden (TMB) has been strongly correlated with clinical responses in patients treated with ICIs [34–37]. TMB is a measurement of the number of nonsynonymous mutations carried by tumor cells. Mutations cause an increased expression of neoantigens in the context of HLA class I antigens enhancing the recognition of cancer cells by cognate T cells. This event is crucial for the development of a host immune response and consequently for ICI efficacy [38]. Therefore a high-TMB is expected to identify patients who are more likely to benefit from ICI-based immunotherapy because of the increased recognition and successive destruction of tumor cells by cognate T cells unleashed by ICIs. Nevertheless, also patients with a high-TMB cancer may not respond to ICIs [38] questioning its role as a predictive biomarker. BCCs are reported to carry a high TMB (65 mutations/Mb), most likely due to the UV signature [39]. In a recent study, Goodman et al. showed that the median TMB for 9 BCC samples and 1637 samples from other type of malignancies was 90/Mb and 4/Mb, respectively [26]. In addition, in two BCCs, Ikeda et al. showed the presence of amplification of the 9p24.3-9p22.2 region which contains the PD-L1, PD-L2 and JAK2 genes [22]. Of interest, PD-L1, PD-L2 and JAK2 amplification is a characteristic of Hodgkin lymphoma, which is exquisitely sensitive to nivolumab [40–42]. Noteworthy, three out of four BCC patients treated with nivolumab presented an objective and durable tumor response [22, 26]. In the patient we have described, we did not investigate the TMB or presence of amplification of the 9p24.3-9p22.2 region. Conversely, we focused our studies on immune cell infiltrate as well as on HLA class I antigen and β2-m expression by tumor cells. Both lack of HLA class I antigen expression and low number of activated cytotoxic T cells (CD8+/ Granzyme B+) can justify the lack of a clinical response to nivolumab. Indeed, HLA class I antigen down-regulation is associated with a decreased recognition of cancer cells by cognate CD8+/Granzyme B+ T cells [43]. HLA class I down-regulation is widely recognized as a mechanism of tumor immune escape and it has been associated to cancer immunotherapy resistance [44]. In BCC, HLA class I antigen down-regulation is associated with a paucity of infiltrating CD8+ T cells [45]. Our data are in line with these findings. In addition, we did not detect β2-m expression in BCC cells. β2-m plays a crucial role in HLA class I antigen expression [46]. No epigenetic alterations have been described for β2-m. Therefore a lack of β2-m expression might reflect mutations in β2-m genes. β2-m truncating mutations have been documented as a mechanism of resistance to anti-PD-1 therapy in melanoma [47]. However we did not perform a genetic analysis of β2-m and additional studies are needed to define β2-m mutations in BCCs. We do not know whether the lack of HLA class I antigen and β2-m expression by tumor cells reflects an escape mechanism to nivolumab of BCC cells or whether nivolumab administration has facilitated the outgrowth of dormant tumor cells not expressing HLA class I antigen expression and subjected to the selective pressure of T cells unleashed by nivolumab.

In some cases HLA class I expression can be restored by interferon gamma (IFNγ) released by infiltrating T cells [48]. Alterations in the IFNγ pathway genes can be responsible of ICI resistance [49, 50], but to the best of our knowledge no information about IFNγ pathway genes is at present available for BCCs. In the patient we have described we hypothesize that HLA class I down-regulation cannot be restored by IFNγ because of the association between lack of β2-m expression and irreversible mutations of β2-m gene. Lastly, we also showed that a BCC tumor is infiltrated by a higher number of negative regulatory immune cells which might also impair the activity of CD8+/ Granzyme B+ T cells [51], undermining the efficacy of PD-1 blockade. All these lines of evidence highlight the potential overlapping of different immunoescape mechanisms. Further studies are warranted in order to elucidate which alteration or spectrum of alterations might be useful to predict the response to ICI-based immunotherapy or which combination of different therapeutic agents might overcome tumor resistance. In BCC Walter et al. showed that the treatment with imiquimod, a toll-like receptor-7 agonist clinically approved for BCC treatment, enhanced HLA class I antigen expression and CD8+ T cell infiltration [45]. Similarly, Otsuka et al demonstrated that the administration of a SHH inhibitor up-regulated HLA class I antigen expression on BCC cells as well as infiltration of CD4+, HLA-DR-class II, and CD8+ cells [52]. These results all together provide the rationale to test combinatorial strategies including ICIs in combination with other immunomodulatory agents and/or targeted agents for BCC treatment.

## Conclusion

Immunotherapy with ICIs is completely revolutionizing the clinical approach to patients with different types of malignancies such as melanoma, NSCLC, renal cell carcinoma, head and neck squamous cell carcinoma, and microsatellite instability-high and mismatch repair-deficient cancers. In addition, several immunotherapeutic agents, alone or in combination, are curently being investigated in other types of solid tumors. In the patient we have described, treatment with anti-PD-1 nivolumab did not inhibit BCC development and relapse. This lack of clinical efficacy was associated with a “cold” tumor microenvironment characterized by the lack of HLA class I antigen subunit expression, low PD-L1 expression and a high regulatory immune cell infiltration.

## Abbreviations

BCC: Basal Cell Carcinoma
CTLA-4: Cytotoxic T Lymphocyte Antigen-4
H&E: Hematoxylin and Eosin
HLA: Human Leukocyte Antigen
ICI: Immune Checkpoint Inhibitor
IHC: Immunohistochemical
mAb: monoclonal antibody
NSCLS: Non-Small Cell Lung Cancer
OS: Overall Survival
PD: Progressive Disease
PD-1: Programmed Death-1
PD-L1: Programmed Death-Ligand 1
PFS: Progression Free Survival
PR: Partial Response
PS: Performance Status
SD: Stable Disease
SHH: Sonic Hedgehog Homolog
TMB: Tumor Mutational Burden
β2m: β2-microglobulin
